# Secreted uPAR isoform 2 (uPAR7b) is a novel direct target of miR-221

**DOI:** 10.18632/oncotarget.3516

**Published:** 2015-03-10

**Authors:** Natalie Falkenberg, Nataša Anastasov, Annalisa Schaub, Vanja Radulovic, Manfred Schmitt, Viktor Magdolen, Michaela Aubele

**Affiliations:** ^1^ Institute of Pathology, German Research Center for Environmental Health, Neuherberg, Germany; ^2^ Institute of Radiation Biology, Helmholtz Zentrum München, German Research Center for Environmental Health, Neuherberg, Germany; ^3^ Clinical Research Unit, Department of Obstetrics and Gynecology, Technische Universität München, München, Germany

**Keywords:** therapy, microRNA, miR-222, PLAUR, soluble

## Abstract

miR-221/-222 and components of the urokinase-type plasminogen activator system (uPAS) are associated with metastasis and poor prognosis in breast cancer, including the triple-negative subtype (TNBC). Modification of components of uPAS and involved miRNAs may contribute to targeted therapy for breast cancer patients. miR-221−/−222-overexpressing or miR-221-depleted cells were employed for qRT-PCR and Western blots to show associations of uPAR with miR-221/-222. To substantiate direct targeting of miR-221/-222 within 3′ UTR of the uPAR isoform 2, *in silico* analysesand *in vitro* assays were conducted. Significant associations between miR-221 and uPAR isoform 2 expressions were observed at the mRNA and protein levels in breast cancer cells representing TNBC. For the first time, the uPAR isoform 2 was demonstrated as direct target for miR-221/-222. Inhibition of miR-221 reduced uPAR protein expression and expression of the tumor cell invasion markers vimentin and RHOC. These results demonstrate a direct and positive regulation of the secreted uPAR isoform 2 by miR-221, increasing its protein expression, a prerequisite for malignancy, while the other uPAR isoforms (1, 3 and 4) are indirectly regulated through miR-10b and miR-221/-222. By targeting uPAR isoforms and/or miRNA-221/-222, the diagnosis and therapy of breast cancer, in particular in TNBC, could be significantly improved.

## INTRODUCTION

Since breast cancer is one of the leading death causes among women in the Western world, novel approaches and additional diagnostic biomarkers for a better therapy are needed [1]. Nowadays, targeted therapies are directed against steroid hormone receptors or human epidermal growth factor receptor 2 (HER2 or ErbB2) [1]. The majority of breast cancer patients is treated with systemic therapies, which are often associated with side effects. microRNAs (miRNAs) are highly involved in tumor progression and may act as novel therapeutic biomarkers [2]. miRNAs are small noncoding RNAs (18 to 24 nucleotides) that affect the stability and translation of targeted messenger RNA (mRNA). Thereby, one miRNA may bind to several regions of the same mRNA or of several mRNAs [3]. Depending on their expression levels and function, miRNAs promote tumorigenesis (oncomiRs) or act as tumor suppressors [4]. In breast cancer, the highly homologous miR-221 and miR-222 promote cell proliferation through the EGFR (epidermal growth factor receptor)-Ras-Raf-MAPK/ERK (mitogen-activated/extracellular signal-regulated protein kinase) pathway [5]. PTEN (phosphatase and tensin homolog deleted on chromosome ten), ER (estrogen receptor), PUMA (p53 upregulated modulator of apoptosis), BMF (BCL2 modifying factor) and p27^Kip1^ represent direct targets of miR-221 and miR-222 [6–10]. These miRNAs regulate EMT (epithelial-to-mesenchymal transition) and enhance tumor development and malignancy when overexpressed, in particular in TNBC (triple-negative breast cancer) and are associated with poor prognosis [5]. Tumor cell invasion is regulated by miR-221/-222 through increasing vimentin expression and reducing E-cadherin by targeting *TRPS1* (Trichorhinophalangeal syndrome type 1) gene as well [5]. TRPS1 negatively regulates the EMT inducer Zinc finger E-box-binding homeobox 2 (ZEB2); miR-221/-222, on the other hand, are regulated by the transcription factor FOSL1 on the transcriptional level [5]. These miRNAs are also associated with resistance to the endocrine drug tamoxifen [11]. Nevertheless, more detailed information is needed for understanding the biological link between these miRNAs with target proteins and their role in tumor progression and metastasis. Recently, we have shown that ectopic overexpression of miR-221 or miR-222 elevates breast cancer cell proliferation and invasion and is associated with breast cancer prognosis [12]. Based on *in silico* analyses, we have found that these miRNAs target the spliced and secreted isoform 2 (uPAR7b) of the urokinase-type plasminogen activator receptor (uPAR), inducing upregulation of this uPAR isoform. uPAR7b was first identified by Pyke and colleagues [13] and further analyzed in human airway and peripheral cells by Stewart and Sayers, who designated the exon 7 deletion as alternative exon 7b [14]. The urokinase-type plasminogen activator (uPA) and its receptor uPAR play important roles in tissue reorganization and wound healing; their upregulation enhances cell invasion and metastasis in cancer cells through degradation of the extracellular matrix (ECM) [15]. Binding of (pro)uPA to uPAR initiates a complex signaling cascade in the tumor cell surrounding space leading to activation of several factors such as plasminogen and fibrinogen [15]. uPA inhibitor type 1 (PAI-1) controls the uPA-uPAR complex formation [15]. PAI-1 acts in a dose-dependent way; low PAI-1 concentrations are associated with a favorable prognosis, whereas very high PAI-1 concentrations do enhance the proliferative potential of tumor cells indicating that PAI-1 promotes the tumor cells growth instead of cell invasion to protect them against the proteolytic degradation in the surrounding normal tissue [16]. Post-transcriptional regulation of uPAR through heterogeneous nuclear ribonucleoprotein C (hnRNPC), p53 and HUR has been shown [17–19]. Furthermore, miR-10b expression is associated with uPAR mRNA and protein expression in higher grade gliomas [20]. Here, we demonstrate that miR-221/-222 directly target 3′ UTR of uPAR isoform 2 (uPAR7b) and positively regulate its translation. We have analyzed the association of uPAR7b in endogenously miR-221−/−222-overexpressing and miR-221-depleted TNBC cells. Based on our results, we propose that uPAR isoform 2 and/or miR-221/-222 are promising targets with regard to a tailored and more efficient therapy of breast cancer, in particular of TNBC.

## RESULTS

### miR-221/-222 and uPAR are significantly co-overexpressed in TNBC

**Figure 1:**
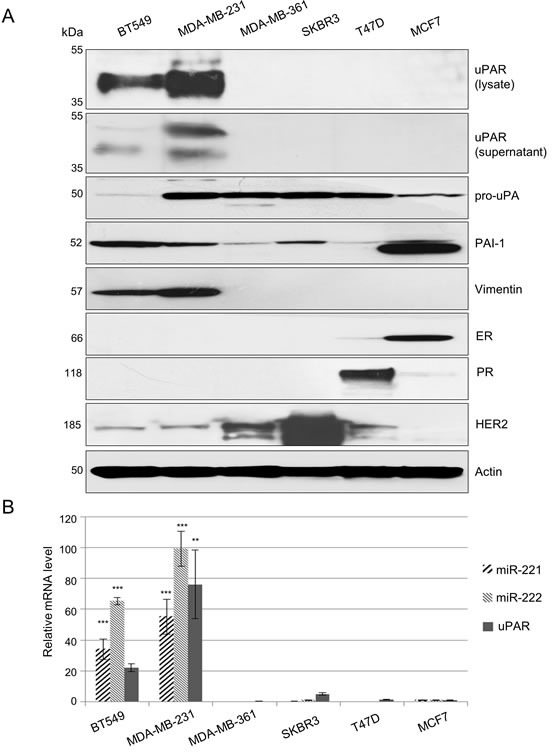
In TNBC, uPAR and miR-221/-222 are strongly co-overexpressed (A) Representative Western blot analyses of uPAR protein expression (in the lysate and supernatant) as well as of the immature form of uPA (pro-uPA), PAI-1, vimentin, estrogen receptor (ER), progesterone receptor (PR), HER2 and actin (as loading control) in the lysates of BT549, MDA-MB-231, MDA-MB-361, SKBR3, T47D and MCF7 breast cancer cells. (B) Quantitative reverse transcriptase-PCR (qRT-PCR) for analysis of miR-221, miR-222 and uPAR expression was performed. Histograms represent relative mRNA expression levels calculated by the 2^−ΔΔCT^ method and normalized to the control (miRNA: RNU43 or mRNA: HPRT1) and to MCF7 cells (calibrator). Data represent means and ± SD (*n* = 3). The Student's *t*-test was used for statistical analysis: * *p* < 0.05, ** *p* < 0.01, *** *p* < 0.001.

For detailed investigation of miR-221/-222 and uPAR in TNBC, model breast cancer cell lines (MDA-MB-231 and BT549) were identified. By immunoblotting, no or weak expression of estrogen receptor (ER), progesterone receptor (PR) and HER2 along with a very strong expression of uPAR and vimentin is demonstrated in MDA-MB-231 and BT549 cells but not in MDA-MB-361, SKBR3, T47D and MCF7 cells (Figure 1A). Protein expression of other uPAS components, such as uPA and PAI-1, were detected at different expression levels of the cell lines used here (Figure 1A). Expression of uPAR was confirmed by qRT-PCR analyses. uPAR mRNA expression is strong in MDA-MB-231 (*p* = 0.004) or BT549 cells but weak in SKBR3 cells or undetectable in MDA-MB-361, T47D or MCF7 cells (Figure 1B). Furthermore, miR-221 and miR-222 are only expressed in TNBC cell lines BT549 and MDA-MB-231 (Figure 1B, each *p* < 0.001).

### uPAR isoform 2 (uPAR7b) sequence analysis defines loss of GPI anchor region

**Figure 2:**
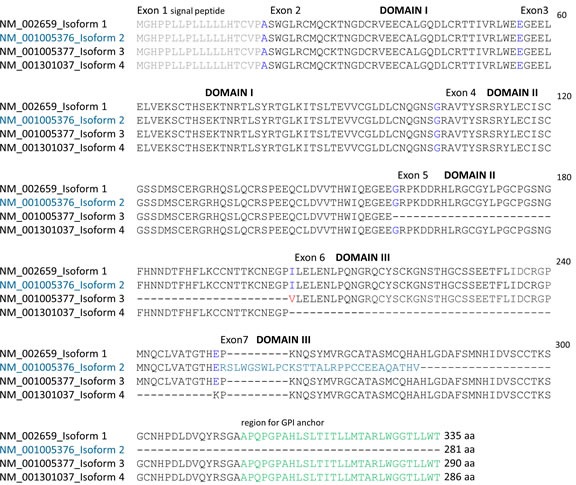
The protein sequence of uPAR isoform 2 (uPAR7b) differs from the protein sequences of uPAR isoforms 1, 3 and 4 Protein sequences of uPAR isoforms 1, 2 (uPAR7b), 3 and 4 were analyzed using CLUSTALW2 software for multiple sequence alignment. The signal peptide (exon 1, grey sequences) is removed and the terminal region at exon 7 (green sequences) is replaced by GPI anchor during processing. Blue amino acids indicate splice junctions, red amino acid indicates valine instead of isoleucine within isoform 3, the alternative sequence instead of exon 7 of uPAR isoform 2 (petrol blue), exons and domains I, II and III (black sequences) are shown. 60 to 335 demonstrate the number of amino acids (aa) of the respective sequence.

**Figure 3:**
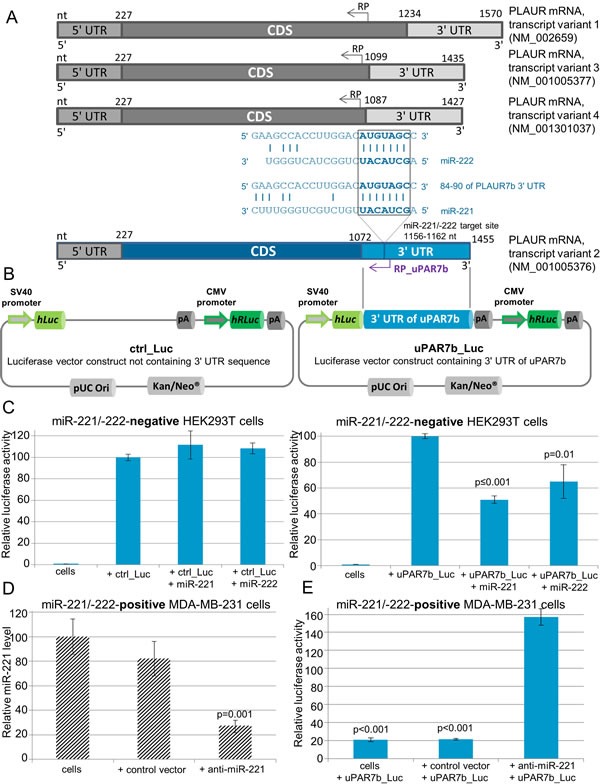
uPAR isoform 2 (uPAR7b) is a direct target of miR-221 and miR-222 (A) A schematic overview of known transcript variants of the human *PLAUR* gene leading to four main isoforms of uPAR is shown. The nt (nucleotides), CDS (coding DNA sequence), 5′ and 3′ UTRs (untranslated region), binding sites for RP (reverse primer) and target site with complementary sequences for miR-221/-222 within 3′ UTR of uPAR isoform 2 (uPAR7b) are indicated. (B) A schematic illustration of control luciferase vector (ctrl_Luc) and miTarget™ 3′ UTR of uPAR7b luciferase vector (uPAR7b_Luc) encoding SV40 promoter, *hLuc* (gene for Firefly luciferase), 3′ UTR of uPAR7b, pA (poly A tail), CMV promoter, *hRLuc* (gene for Renilla luciferase), pUC Ori (origin of replication) and Kan/Neo^®^ (kanamycin/neomycin resistance gene cassette as selection markers) is shown. (C) Luciferase assay for target identification of miR-221/-222 within 3′ UTR of uPAR7b was conducted in HEK293T cells that were transfected with miR-221 or miR-222 overexpression vectors and ctrl_Luc or uPAR7b_Luc. (D) qRT-PCR for analysis of miR-221 expression in MDA-MB-231 cells, which were infected with a control lentivirus (control vector) or infected with lentivirus for miR-221 inhibition (anti-miR-221). (E) MDA-MB-231 cells, cells infected with control vector or with anti-miR-221 encoding lentivirus (anti-miR-221) were transfected with 3′ UTR of uPAR7b luciferase vector (uPAR7b_Luc). Data represent means and ± SD (*n* = 3). The Student's *t*-test was used for statistical analysis.

The gene *PLAUR* encoding the human glycoprotein uPAR is located on chromosome 19q13.1-q13.2 and consists of seven exons, separated by six introns [21]. While exon 1 encodes the 5′ UTR and a signal peptide, exons 2-3, 4-5 and 6-7 encode three homologous Ly-6 antigen/uPAR-like (LU) domains (DI, DII and DIII; Figure 2). According to the NCBI GenBank database, to date four main isoform sequences of uPAR are known, which mostly result from alternative splicing (Figure 2 and 3A). Further alternative splicing events, such as deletion of exon 4 and 5 with prognostic relevance in breast cancer [22] and post-translational modifications, such as glycosylation of uPAR leading to molecular weights between 35 and 60 kDa have been described [21]. The isoform 1 or full-length uPAR (NM_002659) [21] is the longest isoform consisting of three domains and is linked to the plasma membrane by a glycosyl-phosphatidylinositol (GPI) anchor at DIII (Figure 2). When pro-uPA binds to uPAR, it is activated and the uPAR-uPA complex is cleaved close to the GPI anchor and then released into the ECM [15]. The released uPAR-uPA complex has been demonstrated in multiple human diseases, including breast cancer [14]. Compared with full-length uPAR, isoform 2 (NM_001005376) is the shortest isoform arising from alternative splicing of exon 7 within DIII leading to loss of the GPI anchor region and a secreted uPAR (uPAR7b) [13, 14, 23] (Figure 2 and 3A). The physiological role of uPAR7b is not known in detail. Since these modifications do not affect major uPA binding sites within DI and DII, it may interact with and activate uPA and exhibit chemotactic properties leading to enhanced migration and invasion of cancer cells [14]. Isoform 3 (NM_001005377) lacks exon 5 in the coding region, encodes valine instead of isoleucine on the splice site (compared with full-length uPAR) and it is longer than isoform 2 (Figure 2 and 3A). These modifications are supposed to affect glycosylation pattern and folding of uPAR [24, 25]. Isoform 4 (NM_001301037) lacks exon 6 in the coding region and is shorter in comparison to variant 1 but longer than isoform 2 (Figure 2 and 3A).

### miR-221/-222 directly target the uPAR isoform 2 (uPAR7b)

Based on results indicating an association of miR-221/-222 with uPAR7b and due to *in silico* analyses that show a target binding site for miR-221/-222 in 1156-1162 nt of the uPAR7b gene sequence or 84-90 nt within its 3′ UTR (Figure 1 and 3A) [12], luciferase reporter gene assays were conducted to demonstrate direct targeting. We applied a 3′ UTR of uPAR7b luciferase vector (uPAR7b_Luc) that includes the target binding site for miR-221/-222 and a control luciferase vector lacking a 3′ UTR sequence (ctrl_Luc, Figure 3B). To exclude any tumor background that may lead to unspecific binding effects, we ectopically overexpressed miR-221 or miR-222 in the non-transformed cell line HEK293T. When control cells and ectopically miR-221- or miR-222-overexpressing HEK293T cells were transfected with the control luciferase vector, no alteration of luciferase activity was observed (Figure 3C, left). In contrast, when miR-221- or miR-222-overexpressing HEK293T cells were transfected with the uPAR7b_Luc vector, the luciferase activity was significantly reduced (miR-221: p≤0.001; miR-222: *p* = 0.01, Figure 3C, right) in relation to wild-type HEK293T cells transfected with the uPAR7b_Luc vector only, demonstrating a direct targeting of miR-221/-222 within 3′ UTR of uPAR7b. To further investigate this outcome in a breast cancer cell line that endogenously overexpresses miR-221 and miR-222, MDA-MB-231 cells were infected with a control lentivirus (control vector, lacks the sequence for miRNA inhibition) or with a vector, that significantly inhibited miR-221 expression (anti-miR-221, *p* = 0.001, Figure 3D). When MDA-MB-231 cells were treated with anti-miR-221 and transfected with the uPAR7b_Luc vector, the luciferase activity increased in comparison to the MDA-MB-231 cells or cells infected with control vector and transfected with the uPAR7b_Luc vector (each *p* < 0.001, Figure 3E). This outcome underpins the result shown in HEK293T cells and demonstrates 3′ UTR of uPAR isoform 2 as a novel direct target of miR-221/-222.

### miR-221 directly and positively regulates uPAR isoform 2, whereas isoforms 1, 3 and 4 seem to be indirectly regulated by miR-10b and miR-221

**Figure 4:**
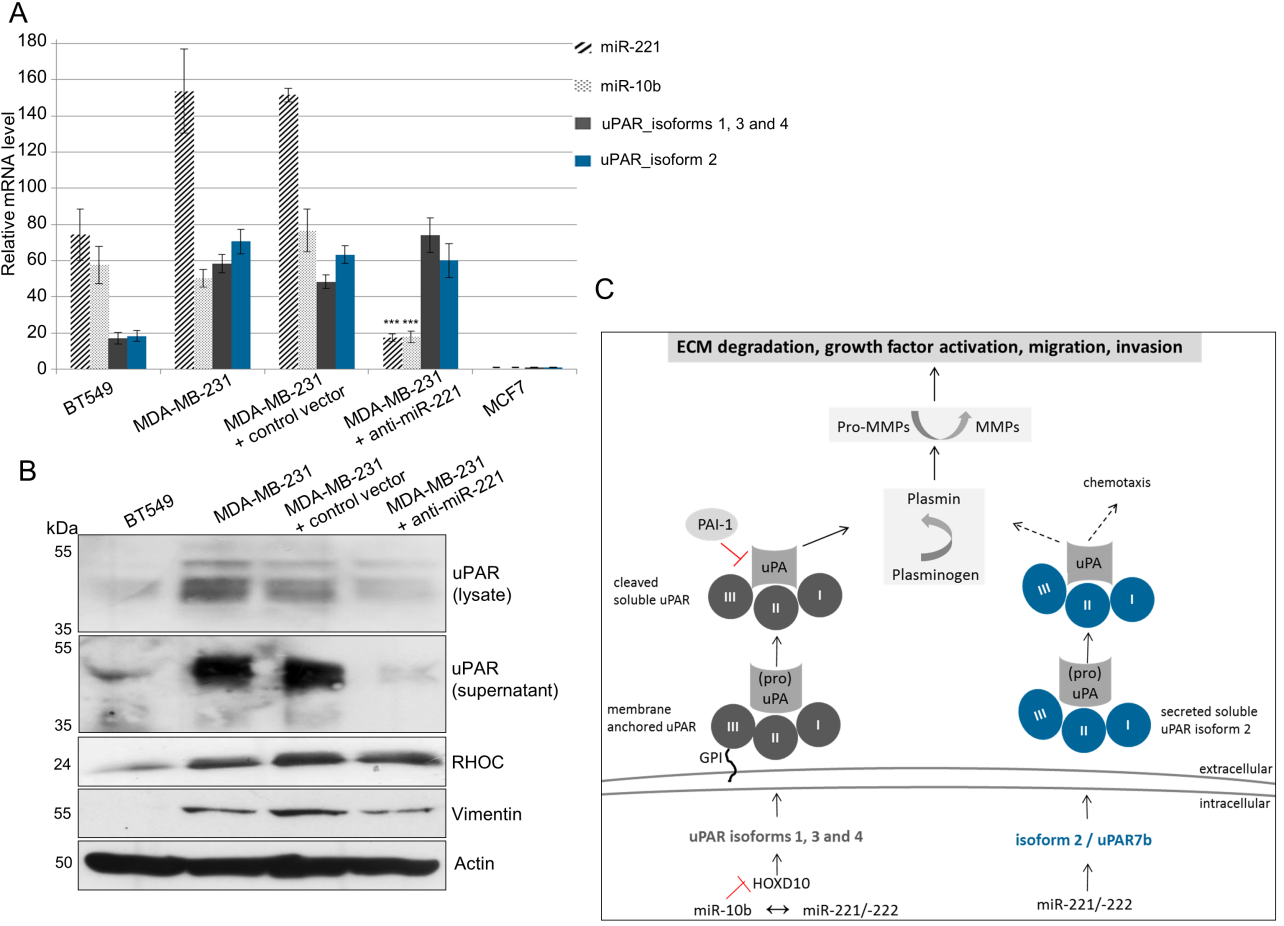
miR-221 directly regulates uPAR isoform 2 (uPAR7b) and indirectly uPAR isoforms 1, 3, 4 and miR-10b (A) qRT-PCR for expression analysis of miR-221 and miR-10b as well as of uPAR isoforms 1, 3, 4 and isoform 2 (uPAR7b). Data represent the means and ± SD (*n* = 3). The Student's *t*-test was used for statistical analysis: * *p* < 0.05, ** *p* < 0.01, *** *p* < 0.001. (B) Representative Western blot analyses of protein expression of uPAR (in the lysate and supernatant) as well as of vimentin, RHOC and actin in the cell lysates are shown. (C) Schematic illustration showing possible molecular mechanisms regulating expression of membrane anchored uPAR isoforms 1, 3 and 4 or the secreted uPAR isoform 2.

In ectopically miR-221- or miR-222-overexpressed HEK293T cells, the miR-221 overexpression stronger and more significantly affected luciferase activity than miR-222 (Figure 3C). Therefore, to investigate the association of overexpressed miR-221 with uPAR in more detail, Western blot and qRT-PCR analyses were performed in those cancer cells, which endogenously overexpress miR-221 or in miR-221-depleted MDA-MB-231 cells. When miR-221 was inhibited by anti-miR-221 in MDA-MB-231 cells (*p* < 0.001, Figure 4A), the mRNA expression of all four uPAR isoforms was almost unchanged compared with non-infected MDA-MB-231 cells (Figure 4A). Regarding protein expressions, a reduction of uPAR in the cell lysate and almost complete reduction of uPAR in the supernatant (indicating secreted uPAR7b) along with reduced levels of the tumor cell invasion marker vimentin following miR-221 inhibition were observed (Figure 4B) while uPAR mRNA levels were not affected. In addition, positive regulation and elevated uPAR protein expression was shown previously using Western blot analysis of ectopically miR-221-overexpressing SKBR3 cells [12] and elevated uPAR mRNA levels of all four isoforms were detected by qRT-PCR in miR-221-overexpressing SKBR3 cells (data not shown).

**Figure 5:**
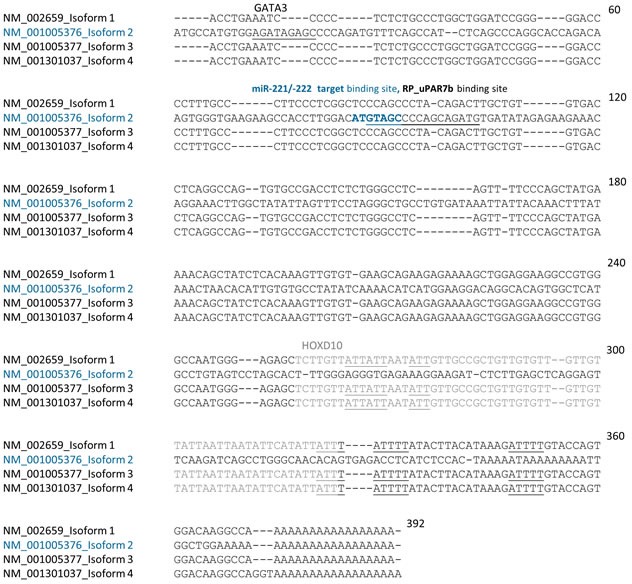
The 3′ UTR of uPAR isoform 2 (uPAR7b) differs from the 3′ UTR of uPAR isoforms 1, 3 and 4 3′ UTR sequences of uPAR isoforms 1, 2 (uPAR7b), 3 and 4 were analyzed using CLUSTALW2 software for multiple sequence alignment. (Potential) binding sites for GATA3 (underlined), for miR-221/-222 (ATGTAGC petrol blue in bold) and reverse primer (RP_uPAR7b, underlined) within 3′ UTR of uPAR7b (NM_001005376) are underlined or indicated. The reverse primer for uPAR isoforms 1, 3 and 4 is not located within the respective 3′ UTR sequences of these isoforms. Potential binding sites for HUR and hnRNPC (underlined 3 to 5 base pairs long gray or black sequences) and for HOXD10 (gray sequence) within 3′ UTR of isoforms 1, 3 and 4 are indicated. 60 to 392 demonstrate the length of nucleotide sequence.

However, mRNA expression of uPAR isoform 2 as well as of the uPAR isoforms 1, 3 and 4 were detected in miR-221-positive cells (Figure 4A). Ma et al., have shown that miR-10b is strongly overexpressed in MDA-MB-231 cells, regulating uPAR expression through translational inhibition of homeobox D10 gene (*HOXD10*) [26]. HOXD10 in turn represses expression of cell migration- and invasion-promoting markers, including (ras homolog family member C) RHOC and uPAR [27]. Therefore, expression of miR-10b was analyzed by qRT-PCR and that of RHOC by Western blotting. While we observe high miR-10b and moderate RHOC levels in miR-221-positive BT549 and MDA-MB-231 cells, the expression levels were significantly (miR-10b: *p* < 0.001, Figure 4A) or slightly (RHOC, Figure 4B) reduced following miR-221 inhibition in MDA-MB-231 cells. To support our theory that uPAR isoform 2 is directly regulated through miR-221 whereas the uPAR isoforms 1, 3 and 4 are indirectly regulated by miR-10b and miR-221 (Figure 4C), we conducted further *in silico* analyses. The induction of isoform 2 following miR-221 overexpression may be additionally controlled through the positive transcription regulator GATA3 (GATA binding protein 3) that is often overexpressed in breast cancer cells [28]. Using *in silico* analysis for putative binding sites for transcription factors [29], we have identified a putative target binding site for GATA3 only within the 3′ UTR of isoform 2 (uPAR7b) and for HOXD10 only within 3′ UTRs of isoforms 1, 3 and 4 (Figure 5). Moreover, HUR, an AU-/U-Rich Element-(ARE)-binding protein and hnRNPC positively regulate uPAR expression [17, 19] and show potential target binding sites only within 3′ UTR of uPAR isoforms 1, 3 and 4 (Figure 5).

## DISCUSSION

TNBC is characterized by strong invasiveness of breast cancer cells and poor prognosis [5]. Here, we investigated the mechanism behind the invasive potential of MDA-MB-231 and BT549 breast cancer cell lines that are representative for TNBC. Several research groups have shown overexpression of miR-221/-222 in MDA-MB-231 cells [5, 30]. Interestingly, we demonstrate strong mRNA and protein expression of invasion-promoting uPAR only in miR-221−/−222-overexpressing MDA-MB-231 and BT549 cell lines. This outcome indicates a physiological association of uPAR and these miRNAs in invasive breast cancer cell lines and confirms our previous observation of uPAR increase following overexpression of miR-221/-222 along with elevated invasion [12].

Based on our previous *in silico* analyses indicating target binding sites of miR-221/-222 within 3′ UTR of uPAR isoform 2 [12], here we performed additional *in silico* analyses for alignments of known uPAR isoforms. To date, four main uPAR isoform sequences are known that result in several splice variants due to post-transcriptional modifications [14]. Different uPAR isoforms have been shown to be of clinical relevance in many tumor entities including breast cancer [14, 22, 23]. Based on protein sequence alignments of the four main uPAR isoforms, we demonstrate that the respective sequences of isoforms 1, 3 and 4 are very similar and are associated to the membrane at the extracellular site by a GPI anchor; in contrast uPAR isoform 2 contains an alternative exon 7b and lacks this GPI anchor (Figure 2 and 4C). Usually, uPAR binds to uPA and the complex is specifically cleaved near the GPI anchor in order to be released into the extracellular space and to degrade ECM and promote cancer cell invasion [15]. uPAR isoform 2 is not associated to the membrane by the GPI anchor and may be (directly) secreted into the extracellular space to enhance malignancy of cancer cells [14].

Using luciferase reporter gene assays in ectopically miR-221- or -222-overexpressing HEK293T cells as well as in endogenously miR-221−/−222-overexpressing or miR-221-depleted MDA-MB-231 breast cancer cells, we proved our previous observation demonstrating direct targeting of miR-221/-222 within 3′ UTR of uPAR isoform 2. Remarkably, the reduction of luciferase activity in MDA-MB-231 cells was much stronger than in ectopically miR-221-overexpressing HEK293T cells. MDA-MB-231 cells not only overexpress miR-221 but miR-222 as well, which is highly homologous to miR-221 and shows the same seed sequence within 3′ UTR of uPAR7b [12]. Therefore, we suggest that binding of endogenous miR-222 to 3′ UTR of isoform 2 additionally decreased the luciferase activity in MDA-MB-231 cells. Based on these findings, we are the first to demonstrate direct binding of miR-221 within 3′ UTR of uPAR isoform 2.

To further explore the association of miR-221 and uPAR isoforms, we performed expression analyses of relevant mRNAs, microRNAs and proteins. Following miR-221 inhibition in MDA-MB-231 cells, reduced expressions of miR-10b along with reduced protein levels of uPAR, RHOC and the tumor cell invasion marker vimentin were observed, while the mRNA levels of all four uPAR isoforms were almost not affected indicating positive regulation of uPAR translation through miR-221. Reduced invasion of MDA-MB-231 cells following miR-221/-222 inhibition has already been shown [31] and is in accordance with our data. Co-overexpression of miR-221 and miR-10b in metastatic thyroid carcinoma cells has been shown by others [32, 33], indicating an association of both miRNAs, while data concerning expression of these miRNAs in breast cancer or TNBC are scarce and inconsistent [34, 35]. Nevertheless, our results indicate that uPAR isoforms 1, 3 and 4 may be indirectly regulated by miR-10b, HOXD10 and miR-221/-222 (Figure 4C and Figure 5), whereas the secreted uPAR isoform 2 (uPAR7b) is directly dependent on miR-221/-222 expression (Figure 3C,) and probably on the positive transcription regulator GATA3 (Figure 5).

Mostly, miRNAs bind their target site in UTRs of mRNA, leading to translational repression, however Vasudevan reports that there is an emerging body of evidence demonstrating translation upregulation mediated by miRNAs [36]. This positive regulation seems to be target specific, may occur under specific conditions or together with co-factors, such as hnRNPC or through ARE-binding proteins including HUR [36]. Furthermore, regarding miR-221/-222, a positive regulation of β-catenin levels through miR-221/-222 has been shown in breast cancer cells [37].

At present, the physiological role of isoform 2 (uPAR7b) is not known in detail, but since it is overexpressed in several cancers and cancer cell lines [13, 14, 23], we hypothesize that miR-221−/−222-overexpressing cancer cells potentiate their malignant capacity through expression of a GPI-anchor-independent uPAR isoform that i) is C-terminally truncated (due to alternative splicing within exon 7 leading to GPI loss), therefore ii) it does not need to be additionally cleaved at the membrane and iii) still possesses chemotactic and ligand-binding properties (Figure 4C). Although further investigations are strongly needed to clarify the association of miR-10b, miR-221/-222 with uPAR isoforms, our study emphasizes the crucial role of miR-221/-222 and uPAR (isoform 2) as invasion-promoting oncomiR/receptor complex. These targets may be promising candidates as predictive, prognostic and/or therapeutic biomarkers, in particular in the aggressive and hard to treat TNBCs.

## METHODS

### Cell culture

The following human breast cancer cells lines MDA-MB-361 (HTB-27), SKBR3 (HTB-30), T47D (HTB-133) and the human embryonic kidney cells HEK293T (CRL-1573) were acquired from American Type Culture Collection (ATCC) and MCF-7 (ACC115) cells from German Collection of Microorganisms and Cell Cultures, DSMZ). The BT549 and MDA-MB-231 cell lines are a kind gift from Prof. M. Schmitt, Clinical Research Unit, Department of Obstetrics and Gynecology, Technische Universität München). The BT549, T47D and MCF7 cells were maintained in RPMI 1640 with GlutaMAX (Rosewell Park Memorial Institute medium) supplemented with bovine insulin (10 μg/μl, Sigma, St. Lois, MO, USA). The SKBR3, MDA-MB-361 and HEK293T cells were maintained in DMEM with GlutaMAX (Dulbecco Modified Eagles medium) that was additionally supplemented with non-essential amino acids (Life Technologies, Darmstadt, D) for cultivation of MDA-MB-231 cells. Both media were supplemented with 10% fetal calf serum (FCS, Invitrogen, Carlsbad, CA, USA) and 0.25% each of penicillin and streptomycin (Life Technologies, Darmstadt, D). The cells were maintained in a water humidified 37°C incubator with 5% CO_2_.

### Transfection and infection of cells

For miR-221 or miR-222 overexpression, 2 × 10^5^ HEK293T cells were transfected with 2 μg plasmid (PMIRH221PA-1-GVO-SBI or PMIRH222PA-1-GVO-SBI, System Biosciences, Mountain View, CA, USA) with Lipofectamine 2000 transfection reagent and OptiMEM (both from Invitrogen, Carlsbad, CA, USA) according to manufacturer's instructions. The transfections were performed in triplicates.

A total of 2 × 10^5^ endogenously miR-221−/−222-overexpressing MDA-MB-231 cells was infected with lentiviral particles for inhibition of miR-221 (anti-miR-221) as described [12]. Replication-defective lentiviral particles were produced by co-transfection of HEK293T cells with the packaging plasmids pMDLg/pRRE, pRSV and the lentiviral expression vector for miR-221 inhibition (MZIP221-PA-1-GVO-SBI, System Biosciences, Mountain View, CA, USA). The lentiviral transduction vector pGreenPuro (control vector, lacks the sequence for miRNA inhibition or overexpression, System Biosciences, Mountain View, CA, USA) was used as an additional control. Three independent infections were conducted.

### RNA isolation

Total RNA was isolated from each of the breast cancer cell lines used in this study. The cells were centrifuged at 1500 rpm for 5 min and washed with 1 ml Dulbecco's phosphate-buffered saline without MgCl_2_ and CaCl_2_ (Invitrogen, Carlsbad, CA, USA). Small RNAs were isolated from the cells using the mirVana miRNA isolation kit (Applid Biosystems, Foster City, CA, USA) following the protocol for total RNA isolation as described [12, 38].

### TaqMan assays and data analysis

Quantitative reverse transcriptase-PCR (qRT-PCR) for analysis of miR-221 and miR-222 expression was performed as described previously [12]. For the analysis of miR-10b expression, following TaqMan-miRNA assay 4427975 and Assay ID 002218 (Applied Biosystems, Life Technologies, Darmstadt, D) were applied. For the generation of cDNA of uPAR following reverse primer (RP) [14]: 5′ TGGGTGGTTACAGCCACTTT 3′ and for uPAR isoform 2/uPAR7b_RP [14]: 5′ ATCACATCTGCTGGGGCTAC 3′ and QuantiTect Reverse Transcription Kit (Qiagen, Hilden, D) according to the manufacturer's instructions were applied. Quantitative PCR was conducted in triplicates by using TaqMan probes (Applied Biosystems, Life Technologies, Darmstadt, D). The mRNA expression levels were calculated by the 2^−ΔΔCT^ method and normalized to the control (miRNA: RNU43 or mRNA: HPRT1) and to MCF7 cells (calibrator) as described previously [38].

### Western Blot analysis

Isolation of proteins, SDS-PAGE and immunoblotting were performed in triplicates as described previously [39]. Following antibodies were used for Western blot detecting uPAR (antibody specifically targets IID7 of uPAR and detects all uPAR isoforms containing the domain II (DII) [40]), uPA (ab24121) can detect (depending on the cell line) the immature pro-uPA (at 50 kDa) and the mature uPA (at 34 kDa), PAI-1 (ab31280, Abcam, Cambridge, MA, USA), vimentin (3390), progesterone receptor (PR, 3157), RHOC (3430, Cell Signaling Technology, Beverly, MA, USA), estrogen receptor (ER, sc-8002, Santa Cruz Biotechnology, Heidelberg, DE), HER2 (A0485, Dako, Glostrup, DK), and actin (A5441, Sigma, St. Louis, MO, USA) as loading control in BT549, MDA-MB-231, MDA-MB-361, SKBR3, T47D and MCF7 breast cancer cells. uPAR was also detected in the cell supernatants whereas all the other proteins were detected in the cell lysates.

### *In silico* analyses

For *in silico* analyses, GenBank sequences of uPAR isoform 1 (NM_002659), isoform 2 / uPAR7b (NM_001005376), isoform 3 (NM_001005377) and isoform 4 (NM_ 001301037) were used. Respective protein or 3′ untranslated region (UTR) sequences were analyzed using CLUSTLW2 software (http://www.ebi.ac.uk/Tools/msa/clustalw2/) for multiple sequence alignment. The analysis for putative binding sites for transcription factors was performed as published by Messeguer and colleagues [29].

### Luciferase reporter gene assay

A total of 2 × 10^5^ HEK293T (ectopically miR-221- or miR-222-overexpressing) or MDA-MB-231 cells and controls were transfected in triplicates with 200 ng luciferase reporter gene vector (uPAR7b_Luc; HmiT013205-MT01) or with 200 ng control luciferase vector (ctrl_Luc; CmiT000001-MT01, vector lacks a 3′ UTR and corresponding miRNA binding site; both vectors from GeneCopoeia™, Rockville, MD, USA), Lipofectamine 2000 transfection reagent and OptiMEM (both from Invitrogen, Carlsbad, CA, USA) according to manufacturer's instructions. After 48 h, the luciferase activity was analyzed in triplicates using the Luc-Pair^TM^ miR Luciferase Assay (GeneCopoeia) according to the manufacturer's instructions.

### Statistical analyses

Means of three experiments were analyzed by the Student's *t*-test. Statistical significance was defined as *p*-value less than 0.05 as follows: * *p* < 0.05, ** *p* < 0.01, *** *p* < 0.001.
